# Online Flutracking Survey of Influenza-like Illness during Pandemic (H1N1) 2009, Australia

**DOI:** 10.3201/eid1612.100935

**Published:** 2010-12

**Authors:** Sandra J. Carlson, Craig B. Dalton, David N. Durrheim, John Fejsa

**Affiliations:** Author affiliations: Hunter Medical Research Institute, Wallsend, New South Wales, Australia (S.J. Carlson, C.B. Dalton, D.N. Durrheim);; Hunter New England Population Health, Newcastle, New South Wales, Australia (S.J. Carlson, C.B. Dalton, D.N. Durrheim, J. Fejsa);; Newcastle University, Newcastle (C.B. Dalton, D.N. Durrheim)

**Keywords:** Flutracking, pandemic, influenza, ILI, surveillance, viruses, dispatch

## Abstract

We compared the accuracy of online data obtained from the Flutracking surveillance system during pandemic (H1N1) 2009 in Australia with data from other influenza surveillance systems. Flutracking accurately identified peak influenza activity timing and community influenza-like illness activity and was significantly less biased by treatment-seeking behavior and laboratory testing protocols than other systems.

A variety of surveillance methods were used to monitor the incidence and severity of influenza A pandemic (H1N1) 2009 in Australia. Severity of illness was measured by number of hospitalizations, intensive care unit (ICU) admissions, and deaths. Influenza disease incidence was monitored through laboratory-confirmed cases, general practitioner sentinel surveillance of influenza-like illness (ILI), emergency department visits for ILI, absenteeism data from large employers, and the Flutracking surveillance system (1).

Flutracking is a national weekly online survey of ILI (completed by >8,000 participating community members each week in 2009); it is the only ILI surveillance system that provides comparable data across Australia’s states and territories. Flutracking integrates participants’ ILI symptom information with their influenza vaccination status (2). Flutracking surveillance has correlated well with other Australian influenza surveillance systems in describing the timing and scale of the 2007 and 2008 seasonal influenza epidemics (3,4). We compared Flutracking data with data from other routine influenza surveillance systems during the 2009 pandemic wave in New South Wales (NSW), Australia’s most populous state.

## The Study

From May 4, 2009, through October 31, 2010, participants received an automatically generated weekly email link to the online questionnaire, which asked whether they had experienced fever or cough and how many days they had been absent from work or normal duties because of these signs (recruitment details in *2,3*; location of participants at www.flutracking.net). Each individual response usually took <15 seconds. Participants who had previously reported not receiving seasonal influenza vaccine were asked whether they had received influenza vaccination in the prior week during each weekly survey. If they answered yes, the question was automatically deleted from their subsequent weekly surveys. Participants were permitted to enroll at any time during the surveillance period. Participants could respond on behalf of household members, and children >12 years of age could complete their own survey. During online enrollment, participants provided the following information: their month and year of birth; whether they had received a seasonal influenza vaccine in the preceding year; whether they worked face to face with patients in hospitals, nursing homes, doctors’ clinics, or as community health workers; and their residential postal code.

The weekly proportion of participants with ILI signs or symptoms was calculated as the proportion of participants for that week who reported both fever and cough within the previous 7 days. These proportions were compared with influenza activity recorded in 2009 by other established New South Wales influenza surveillance systems, i.e., number of patients who visited emergency departments with ILI symptoms (5), laboratory-confirmed influenza A antigen tests (PCR and direct immunofluorescence) (5), Google Flu Trends data (aggregated Google search data used to estimate current influenza in Australia) (6), workplace absenteeism data (5), and Australian Sentinel Practice Research Network (ASPREN) general practice ILI data (7).

Surveillance data were compared with data from 2007 and 2008. NSW was selected because no other states had sufficient Flutracking participants in 2007 and 2008 to allow year-to-year comparisons. The number of NSW participants who completed >1 survey in the 2009 Flutracking surveillance system was 3,447.

The concordance across NSW influenza surveillance systems was high for ILI peak weeks during the past 3 years. During 2009, Flutracking, laboratory influenza notifications, and Google Flu Trends peaked 1 week before emergency department ILI, workplace absenteeism, and ASPREN ILI surveillance (Table).

**Table:** Peak ILI attack week and attack rates across influenza surveillance systems in New South Wales, Australia, 2007–2009*

Surveillance system/weekly measure used	Peak week of ILI (week ending)				Peak ILI/influenza-related values		
	2007	2008	2009		2007	2008	2009
Flutracking, fever and cough rate, %	Aug 5	Aug 24	Jul 12		9.4	5.8	6.8
No. laboratory notifications	Aug 5	Aug 31	Jul 12		133	69	1,167
No. ED ILI visits	Aug 19	Aug 31	Jul19		374	170	1,024
Google Flu Trends							
Influenza-related search term counts	Jul 22	Aug 31	Jul 12		1,933	1075	1,022
Workplace absenteeism, weekly rate, %	Jul 15	ND	Jul 19		1.5	ND	1.4
ASPREN, ILI/1,000 consultations, %	Aug 12	Sep 7	Jul 19		73.7	62.8	74.3

*ILI, influenza-like illness; ED, emergency department; ND, no data collected; ASPREN, Australian Sentinel Practice Research Network.

A comparison of the weekly scale of NSW Flutracking fever and cough symptom rates during 2007, 2008, and 2009 showed that the peak attack rate of 6.8% in 2009 was significantly lower than that of 9.4% in 2007 and only slightly higher than the peak rate of 5.8% in 2008 (Figure). However, peak weekly NSW laboratory notifications were almost 9- and 17-fold higher in 2009 than in 2007 and 2008, respectively. Peak emergency department ILI patient visits were almost 3- and 6-fold higher in 2009 than in 2007 and 2008, respectively (Table; Figure).

**Figure:**
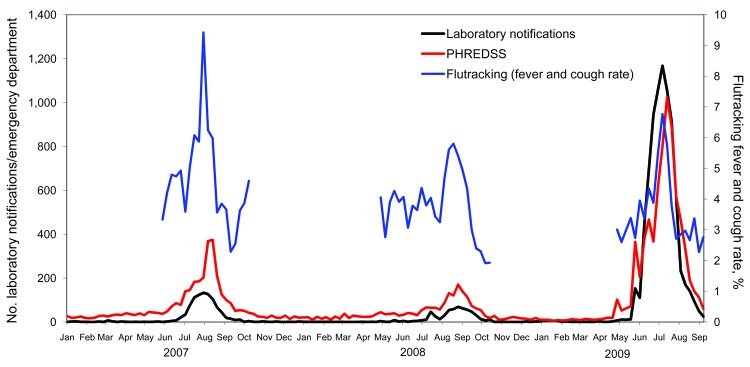
Flutracking fever and cough rates, counts of emergency department visits for influenza, and number of laboratory notifications for influenza, New South Wales, Australia, 2007–2009. PHREDSS, Public Health Real Time Emergency Department Surveillance System**.**

The attack rate pattern for NSW Google Flu Trends data was similar to that of Flutracking; attack rates for 2009 were slightly lower than those for 2008 and about half those of for 2007. ASPREN ILI rates were slightly higher in 2009 than in 2007 and 2008. Workplace absenteeism data demonstrated a slightly more severe influenza season in 2007 than in 2009 (Table).

When the surveillance systems were compared, laboratory notifications and emergency department surveillance appeared to be more affected by health-seeking behavior and changes in physician’s testing protocols and may not have reflected true community ILI rates, in contrast to Flutracking, Google Flu Trends, workplace absenteeism, and ASPREN. Potential biases in laboratory notifications and emergency department surveillance may vary, depending on the pandemic phase. For example, during the protect phase of the pandemic, testing for influenza was recommended only for those admitted to the hospital for ILI or when test results could alter clinical care of a patient. Before the protect phase (during the contain phase), testing for pandemic (H1N1) 2009 virus was conducted to confirm diagnosis for anyone with ILI.

Flutracking’s finding of a 2009 peak ILI rate similar to those of previous years was also consistent with NSW mortality data. The number of NSW deaths attributed to influenza or pneumonia suggested that the 2009 influenza season did not result in excess overall deaths but rather a redistribution of deaths with a relative increase of deaths in younger age groups (8). The low ILI rate found by Flutracking was initially viewed with suspicion because other near real-time surveillance (laboratory notifications and emergency department surveillance) suggested a high pandemic (H1N1) 2009 attack rate compared with rates for previous years. However, Flutracking results were consistent with other pandemic influenza attack rate estimates in NSW and other countries (9–12).

Because Flutracking does not rely on the health sector for ILI or laboratory reporting, it is not biased by changes in testing, treatment seeking, jurisdictional protocols, or resource constraints. Flutracking, Gripenet, and other similar Internet-based surveillance could potentially facilitate near real-time comparison of ILI activity between regional jurisdictions and among countries to assist with monitoring the global spread of influenza (13).

## Conclusions

During the initial pandemic (H1N1) 2009 outbreak, Flutracking demonstrated its ability to accurately identify peak influenza activity timing and the relative magnitude of community influenza activity when compared with influenza tracking efforts in previous years. Its results were also less affected by treatment-seeking behavior and by laboratory testing protocols during different pandemic phases than was health system–based surveillance.
